# Construction of a mineralized collagen nerve conduit for peripheral nerve injury repair

**DOI:** 10.1093/rb/rbac089

**Published:** 2022-10-31

**Authors:** Guman Duan, Chengli Li, Xiaoqing Yan, Shuhui Yang, Shuo Wang, Xiaodan Sun, Lingyun Zhao, Tianxi Song, Yongwei Pan, Xiumei Wang

**Affiliations:** State Key Laboratory of New Ceramics and Fine Processing, Key Laboratory of Advanced Materials of Ministry of Education, School of Materials Science and Engineering, Tsinghua University, Beijing 100084, China; Department of Orthopedics, Beijing Tsinghua Changgung Hospital, School of Clinical Medicine, Tsinghua University, Beijing 102218, China; State Key Laboratory of New Ceramics and Fine Processing, Key Laboratory of Advanced Materials of Ministry of Education, School of Materials Science and Engineering, Tsinghua University, Beijing 100084, China; Department of Orthopedics, Beijing Tsinghua Changgung Hospital, School of Clinical Medicine, Tsinghua University, Beijing 102218, China; State Key Laboratory of New Ceramics and Fine Processing, Key Laboratory of Advanced Materials of Ministry of Education, School of Materials Science and Engineering, Tsinghua University, Beijing 100084, China; Department of Orthopedics, Beijing Changping District Hospital, Beijing 102202, China; State Key Laboratory of New Ceramics and Fine Processing, Key Laboratory of Advanced Materials of Ministry of Education, School of Materials Science and Engineering, Tsinghua University, Beijing 100084, China; State Key Laboratory of New Ceramics and Fine Processing, Key Laboratory of Advanced Materials of Ministry of Education, School of Materials Science and Engineering, Tsinghua University, Beijing 100084, China; State Key Laboratory of New Ceramics and Fine Processing, Key Laboratory of Advanced Materials of Ministry of Education, School of Materials Science and Engineering, Tsinghua University, Beijing 100084, China; State Key Laboratory of New Ceramics and Fine Processing, Key Laboratory of Advanced Materials of Ministry of Education, School of Materials Science and Engineering, Tsinghua University, Beijing 100084, China; Beijing Allgens Medical Science and Technology Co., Ltd, Beijing 100176, China; Department of Orthopedics, Beijing Tsinghua Changgung Hospital, School of Clinical Medicine, Tsinghua University, Beijing 102218, China; State Key Laboratory of New Ceramics and Fine Processing, Key Laboratory of Advanced Materials of Ministry of Education, School of Materials Science and Engineering, Tsinghua University, Beijing 100084, China

**Keywords:** nerve guidance conduit, collagen, mineralized collagen, peripheral nerve regeneration

## Abstract

A new nerve guidance conduits (NGCs) named MC@Col containing Type I collagen (Col) and mineralized collagen (MC) was developed, enhancing mechanical and degradation behavior. The physicochemical properties, the mechanical properties and *in vitro* degradation behavior were all evaluated. The adhesion and proliferation of Schwann cells (SCs) were observed. In the *in vivo* experiment, MC@Col NGC and other conduits including Col, chitosan (CST) and polycaprolactone (PCL) conduit were implanted to repair a 10-mm-long Sprague-Dawley rat’s sciatic nerve defect. Histological analyses, morphological analyses, electrophysiological analyses and further gait analyses were all evaluated after implantation in 12 weeks. The strength and degradation performance of the MC@Col NGC were improved by the addition of MC in comparison with pure Col NGC. *In vitro* cytocompatibility evaluation revealed that the SCs had good viability, attachment and proliferation in the MC@Col. In *in vivo* results, the regenerative outcomes of MC@Col NGC were close to those by an autologous nerve graft in some respects, but superior to those by Col, CST and PCL conduits. The MC@Col NGC exhibited good mechanical performance as well as biocompatibility to bridge nerve gap and guide nerve regeneration, thus showing great promising potential as a new type of conduit in clinical applications.

## Graphical Abstract

**Figure:**
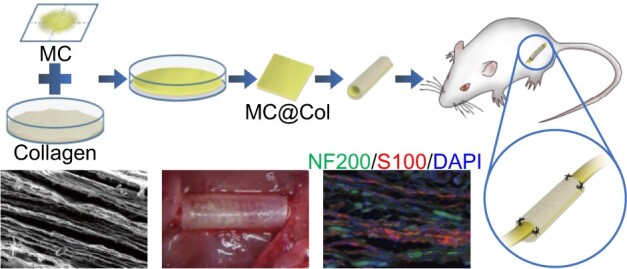

## Introduction

Peripheral nerve injury (PNI) is a quite common clinical injury [1], and it can lead to significant disability [2], affecting around 5 million individuals throughout the world every year [3]. Peripheral nervous system has a greater capacity to regenerate after injury versus central nervous system [4, 5], but it still has limits [6]. Even if the so called ‘gold standard’, autografts still limited by shortage of nerves, donor site morbidity and other potential complication risks [7, 8]. Therefore, scientists have developed nerve graft substitutes, including decellularized allogenic nerves, xenogenic nerves and synthetic nerve guidance conduits (NGCs) [3, 5].

The usage of artificial NGCs in repairing nerve injury originated nearly 100 years ago [9]. The scar formation prevention and reestablishment of regenerative microenvironment for neurite outgrowth were the two main aims provided by NGCs [10]. A range of NGCs have been developed and tested in animals and/or a clinical context, such as collagen (Col) [10], chitosan (CST) [11], polycaprolactone (PCL) [10, 12] and polyglycolic acid [13]. Among them, Col is the most popular biomaterial used both in scientific research as well as clinical products [10].

Col, one of the major structural proteins, has been widely explored as a basic materials potentially in NGCs, because of the biocompatibility, biodegradability and low antigenicity [14, 15]. In different experimental studies, as a nerve conduit material, Col, is believed to facilitate Schwann cells (SCs) migration and neurite outgrowth in the nerve repair process, and Col has been extensively employed to form an outer tubular construct and as a central luminal filler for nerve regeneration [14]. For now, there are a number of Col-based peripheral nerve conduits in the market [14], such as the NeuraGen (Integra LifeSciences, Plainsboro, NJ), the NeuroMatrix Col matrix (Stryker Orthopaedics, Mahwah, NJ) and the Neuroflex Col matrix (Stryker) nerve cuffs [16]. However, there are some characteristics that limited its clinical applications, including poor mechanical strength and too fast degradation [14]. The purpose of this study is to overcome these problems by constructing a new Col-based conduit, which not only has good biocompatibility, but also has appropriate degradation, good mechanical properties and regeneration function.

The calcium ion has been recognized improving peripheral nerve regeneration in some respects [17], which modulate growth cone, motility and turning through the signaling pathway [18]. According to the studies, calcium-mediated signaling pathways participate in the axonal outgrowth, the axonal migration and the axonal extension through Netrin-1 signaling pathway activating [19, 20]. The biomimetic mineralized collagen (MC), as the reservoir of calcium ions, was developed previously in our laboratory and was used in this study [21–24]. It is composed of Col and nano-hydroxyapatite (HA), which the chemical composition and microstructure were similar with the natural material *in vivo* [25].

In this study, a new NGC based on Col and biomimetic MC (MC@Col) was constructed. After evaluating the physicochemical properties and biodegradation, MC@Col conduits were then implanted *in vivo* to bridge a sciatic nerve gap in Sprague-Dawley (SD) rats [26], in comparison with Col, CST and a PCL conduits. Finally, the conduit induced changes of axonal regeneration, the muscle reinnervation and the motor recovery were all evaluated by morphological, histological, electrophysiological and gait evaluations.

## Materials and methods

### Experimental design

**Figure 1.**
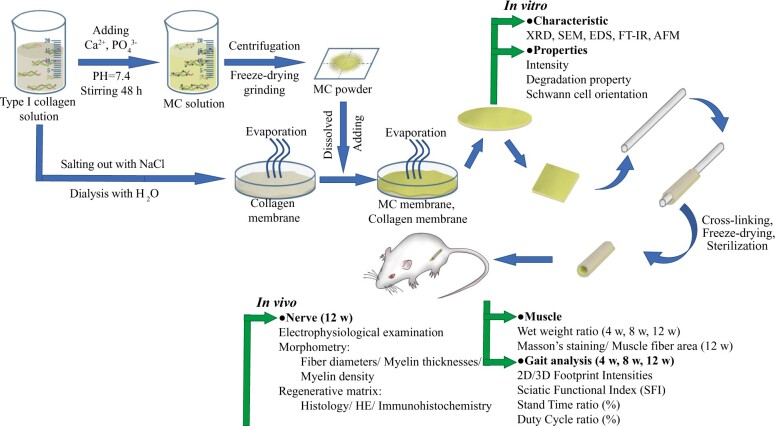
Illustration of study design and the various steps of the comprehensive *in vitro* and *in vivo* analyses. The synthesized membrane were tested *in vitro*, and the conduits were shaped in multi-layered MC@Col conduit for *in vivo* surgery. MC, mineralized collagen; AFM, atomic force microscopy; FTIR, Fourier-transform infrared spectrometer; EDS, energy dispersive spectrometer; h, hours; w, weeks.

Figure 1 illustrated our experimental design and the main elements of *in vitro* and *in vivo* studies.

### Fabrication of MC@Col conduit

The MC@Col conduit was basically fabricated with Col and biomimetic MC. MC powder was synthesized according to the method reported previously [22]. First, the calcium ion and phosphate anion were dropped into acidic Col solution (the calcium/phosphorous ratio of which was 1.67). Then with a constant 48 h of stirring, the pH value of this solution was then adjusted to 7.4. The MC deposition was appeared gradually, and then it was collected through centrifugation, freeze-dried and grinding in sequence. Type I Col was obtained from bovine Achilles tendon using a protocol described previously [27]. Briefly, the tendon was dissolved in HCl solution, centrifuged and filtered. The precipitation was induced by NaCl, and it was redissolved in HCl solution. Later, a gelatinous Col was collected in the dialysis bags from the acid-soluble mixture (provided by Beijing Allgens Medical Science and Technology Co., Ltd., Beijing). This extracted Col was then put into a container for air dry. Previously prepared MC powder was added on the top of the Col membrane (0.5–1 g/cm^2^) before it was completely dry. Furthermore, this membrane was wrapped into multi-layered conduit (Fig. 1) and crosslinked by 1-ethyl-3-(3-dimethylaminopropyl)carbodiimide. Finally, these membranes and conduits were sterilized once before test *in vitro* and *in vivo*.

### Physicochemical characterizations of the MC@Col conduit

#### Molecular structures and surface morphologies

The structure of the MC conduit was analyzed with X-ray diffraction (XRD) and Fourier-transform infrared spectroscopy (FTIR). The XRD results were used to determine the structure and crystallinity of the materials [25]. The MC@Col and Col tubes were ground into powder and were analyzed with a D/max-2550 diffractometer (Rigaku, Tokyo, Japan) using Cu/Kα radiation source (*λ *= 1.54 Å) over the 2*θ* range of 3°–90° with a scan speed of 4°/min. The Infrared spectra were detected by a VERTEX 70 V system (Bruker Company, Germany). The MC@Col and Col conduits were ground and mixed with KBr, and then were pressed into pellets for FTIR spectroscopy analysis. The infrared absorption spectrum of the samples was measured range from 400 to 4000 cm^−1^.

The micromorphologies of two conduits were observed using a scanning electron microscope (SEM), involving the outer surface, the inner surface and the fresh fractured side surface. The samples were fixed on a specimen stage using a conductive adhesive strip and sputter-coated with platinum alloy in EM ACE600 (Leica, Germany), and then characterized the morphology by the GEMINISEM 500 microscope (Carl Zeiss, Germany). Energy-dispersive X-ray spectroscopy (EDS) was also applied to determine the elemental proportions and distribution of Ca and P of different sites of specimens.

Both the mechanical property and nanostructure at the microscale level of the two samples (MC@Col and Col) were characterized through the quantitative nano-mechanical atomic force microscope (AFM) [28]. For different conduits, the micromorphology, surface stiffness and roughness were all analyzed by AFM (Dimension ICON, Bruker, USA). Specimens were detected in peak force tapping mode, and the scan rate was set as 1 Hz. In each sample, a 3 μm × 3 μm was set as the scan size. As a general quantitative parameter measuring roughness [29], the R_q_ (the root mean square average of the roughness profile ordinates) was further calculated in different samples. About the micro-mechanical properties, the Young’s modulus were then determined using the force-indentation plots and the Hertz model [8]. The software NanoScope Analysis 1.8 (Bruker, USA) was used in data processing.

#### Physical properties

For the measurements of the macro-mechanical properties of the Col membrane and MC@Col membrane, the tensile strength of the specimens (10 mm in width, 40 mm in length, *n* = 5) were measured both in wet (tested after 30 min soaking in normal saline) and dry conditions [30]. The testing results were obtained using two universal materials testing machines, one (EZ-LX HS 1KN; SHIMADZU, Japan) for wet condition and another (AGX-V 20KN; SHIMADZU, Japan) for dry condition. The crosshead speed was set at 5 mm/min. The tensile strengths and modulus of the specimens were then computed using a TRAPEZIUM X-V software (SHIMADZU, Japan). These were repeated five times in each kind of sample for statistical analysis. Besides, the radial compressive resistance of four types of conduits were also evaluated by a universal material testing machine (UTM4204, 20 kN; SUNS, China). The compressive force corresponding to the diameter of conduits reduced by 50% were recorded for comparison, and this kind of measurements were repeated for three times.

#### *In vitro* biodegradation

It has been reported that the biodegradability of a nerve scaffold is important in the peripheral nerve reconstruction [31]. In this study, the degradability *in vitro* were analyzed by incubating the membranes in phosphate buffer saline (PBS) (1 mol/l, pH 7.4) and lysozyme (0.5 mg/ml; Biodee Biotechnology, China) at indoor temperature (37°C) with constant shaking at 60 rpm using the water baths shaker (WE-1, Honour Instrument, China) [32]. While incubating solution was renewed in every 3 days, the samples were taken out and weighted at specified time after washed by distilled water and lyophilized. Based on the previous studies, the extent of degradation was measured by mass loss of samples through the following equation [30]: *D* = (*W – W*_0_)/*W*_0_ × 100%, where *W*_0_ is the initial weight and *W* is the weight after incubation for 3, 7, 14, 21 and 28 days. These were repeated five times in each group for statistical analysis.

### *In vitro* biocompatibility evaluations

#### *In vitro* cell culture of rat SCs

The cells were purchased from National Infrastructure of Cell Line Resource (RSC-96, 3111C0001CCC000664; China) and were cultured in Dulbecco’s Modified Eagle Medium (Gibco, USA), in which the percentage of fetal bovine serum (Gibco, USA) and penicillin–streptomycin (Gibco, USA) solution were 10% and 1%, respectively. The cells were then cultured at 37°C under a humidified 5% CO_2_ atmosphere. Three types of materials were then included in the co-culture, and two groups (MC@Col and Col membranes) were prepared as described above. As a control, HA@collagen (HA@Col) membrane was synthesized similarly with MC@Col but the MC particles was replaced by HA as the same amount of calcium phosphate. The rat SCs (RSCs) were seeded on the surface of these three different materials (MC@Col, Col and HA@Col) at a density of 2 × 10^4^ cells/well (48-well tissue culture plate) and the growth medium was refreshed every 2 days. After culture for 1, 3, 5 and 7 days, the samples were incubated with 10% Cell Counting Kit-8 (CCK-8, Dojindo, Japan) working solution in cell culture medium keep in the dark at 37°C for 2 h. Finally, 100 μl of the culture media were transferred to another 96-well plate, and the absorbance was tested at 450 nm by using a microplate reader (EnSpire Multimode Plate Reader, PerkinElmer, USA) [33]. This was repeated five times in each group at each time point for statistical analysis.

#### Immunofluorescence staining

After 1, 3, 5 and 7 days, the co-culture RSCs were collected and washed three times with the PBS (0.01 M). After managed by 4% paraformaldehyde (20 min), 0.1% Triton X-100 solution (5 min), PBS washing and 1% bovine serum albumin incubation (Sigma-Aldrich, USA) (30 min) in turn, actin filaments and nuclei were stained with Rhodamine-Phalloidin (1:300; Cytoskeleton, USA) and 5 μM SYTOX green nucleic acid stain (Invitrogen, USA), respectively [33]. The images were captured by laser confocal scanning microscopy (LSM 710, Carl Zeiss, Germany).

#### Morphology of RSCs

The SEM were used in observing the morphology of co-culture RSC96. After gradient dehydration in ethanol from 30 to 100%, the samples were immersed in tert-butanol, dried through freeze-drying, coated with a layer of platinum film and observed through SEM finally.

### *In vivo* implantation

#### Animals and surgical procedures

In this study, the animals’ raising and operating guidelines were approved by the ethics committee of the Laboratory Animal Research Center of Tsinghua University (Approval document number: 19-WXM2). A total of 75 adult male SD rats weighing 200–220 g (housed in the Laboratory Animal Research Center of Tsinghua University) were divided into five animal groups randomly, including autologous nerve graft transplanted group (Autograft group, *n* = 15), MC@Col nerve conduit group (MC@Col group, *n* = 15), Col nerve conduit group (Col group, *n* = 15), CST nerve conduit group [34] (CST group, *n* = 15) and PCL nerve conduit group [3] (PCL group, *n* = 15). The rats were first anesthetized through intraperitoneal injection (2% sodium pentobarbital solution, 30 mg/kg). After skin antisepsis, the sciatic nerve of the right side was exposed and excised, leaving a 10-mm-long nerve gap after the nerve ends retraction for the conduit groups. The defects were bridged with four types of conduits mentioned above with epineurial sutures (8–0 nylon, LINGQIAO, Ningbo Medical Needle, China). For the Autograft group, 10-mm-long transected nerve were flipped and turned 180° around its longitudinal axis, and then performing orthotopic implantation subsequently through epineurial sutures. After the wound closed, all the animals were kept in a standard environment. The individual rats were harvested at 4, 8 and 12 weeks after surgery for the later examinations.

#### Immunofluorescence and hematoxylin–eosin staining of regenerated nerves

Twelve weeks after surgery, the right operated sciatic nerves were harvested, and the samples were then cut into sections longitudinally through a freezing microtome (CM1950, Leica, Germany). With reference to the studies previously published [34], these sections were later stained with two primary antibodies: anti-neurofilament 200 antibody (NF200; 1:200, mouse IgG_1_, Abcam) for regenerated axons and anti-S100 antibody (S100; rabbit polyclonal, Abcam) for SCs. The secondary antibody Alexa 488 (goat anti-mouse IgG_1_, Thermofisher, USA) and Alexa 594 (goat anti-rabbit polyclonal, Thermofisher, USA) as well as DAPI were used to stain the sections later. In contrast to the immunofluorescence-stained slices, some of the neighbor sections were subjected to hematoxylin–eosin staining. Finally, all the stained sections were viewed through a fluorescence microscope mode or light microscope mode in the Axio-Scan Z1 microscope (Carl Zeiss, Germany), and the images were quantified using the ImageJ software (ver.1.53f, National Institutes of Health, USA).

#### Observation of myelinated axons of regenerated nerves

Based on a previous studies [8], the right sciatic nerves (12 weeks after surgery) were excised from the middle and were fixed in 2.5% glutaraldehyde (4°C for 2 h), 1% osmium tetroxide solution, dehydrated using graded ethanol, embedded in epoxy resin (Epon 812; Fluka, Germany) and cut into semi-thin and ultrathin sections. The semi-thin sections (700 nm) were stained with 1% toluidine blue/1% borax solution and visualized under light microscope (Pannoramic SCAN; 3DHistech, Hungary), and the myelinated axons density was determined from these images. Five individual rats per group were repeated for statistical analysis (*n* = 5) and average density from five random fields for each animal. The ultrathin sections were stained with lead citrate and uranyl acetate, then observed by a transmission electron microscope (TEM) (JEM-1400EX; JEOL, Japan) [8]. These TEM images were used to calculate the thickness of myelin sheaths, the diameter of myelinated nerve fibers, g-ratio-based diameter and g-ratio-based perimeter (the ratio between mean diameter/perimeter of an axon and the mean diameter/perimeter of the fiber including myelin) for evaluating the degree of fiber myelination. Five individual rats per group were repeated (*n* = 5), and for each animal specimen, all the visualized axons from five random TEM sectional images (more than 20 axons each image) were calculated and averaged. The images were quantified using the ImageJ software.

#### Electrophysiological assessment

At 12 weeks after surgery, the right and left sciatic nerve were re-exposed for electrophysiological studies after the motor functional analysis (*n* = 5 per groups) based on the previously published article [34]. The amplitudes and latency of the compound muscle action potential (CMAPs) of the sciatic nerves were recorded by the PowerLab 4SP system (Keypoint 3.02; Denmark) at both the injured side and the normal side of the experimental rats. The ratio of both side of the CMAPs (latency and peak) were calculated and compared between groups subsequently.

#### Reinnervated muscle weight and remodeling of muscle fibers

Studies have shown that the target muscles atrophied after its denervation and gained part of reversion after nerve regeneration [34]. In this part, the degree of the gastrocnemius changes was tested both in gross and histological view and compared within groups to understand the efficiency of re-innervate in different group [8]. At 4, 8 and 12 weeks after surgery, the gastrocnemius muscles harvested from both sides and were immediately weighed (*n* = 5 per group in 12 weeks; *n* = 3 per group in 4 or 8 weeks, respectively). The ratio of the wet weight of the gastrocnemius between both sides, and the ratio of muscle fiber area between both sides were calculated. The detailed procedure referred to a previously studies [8]. The Masson’s trichrome staining images from each animal were obtained from five randomly chosen fields with the Axio-Scan Z1 microscope. Finally, the muscle wet weight ratio and muscle fibers cross-sectional area were calculated by ImageJ software based on previously methods [34].

#### CatWalk gait analysis

In this study, the motor function recovery of sciatic nerves was monitored by the CatWalk XT 10.60 gait analysis system (Noldus, the Netherlands) at 4, 8 and 12 weeks after surgery. The rats (*n* = 5 per group in 12 weeks; *n* = 3 per group in 4 or 8 weeks, respectively) were all tested based on the previously described protocol [34]. During runs, the footprints, stand time and intensities of both sides of experimental rats were recorded for later analysis. To evaluate the functional recovery of the sciatic nerve, the sciatic functional index (SFI) was also calculated according to the following formula [35] where ETS refers to the experimental toe spread, NTS refers to the normal toe spread, EPL refers to the experimental print length, NPL refers to normal print length, EIT refers to experimental inter toe spread, and NIT refers to normal inter toe spread.

SFI = –38.3((EPL–NPL)/NPL) + 109.5((ETS–NTS)/NTS) + 13.3((EITS–NITS)/NITS) – 8.8

### Statistical analysis

Statistical analysis of data was carried out using independent *t*-tests or one-way analysis of variance (ANOVA). When the ANOVA test for multi-group comparisons was significant difference, the Tukey’s HSD *post hoc* test (equal variances) or Dunnett’s T3 *post hoc* test (unequal variances) was conducted for further comparison between two groups. A statistically significant difference was conducted when *P* was < 0.05. All values were reported as mean ± standard deviation (SD) and all statistical analysis was conducted using SPSS 23.0 software (IBM corp., USA).

## Results

### Physicochemical characterization of the synthesized conduits

**Figure 2.**
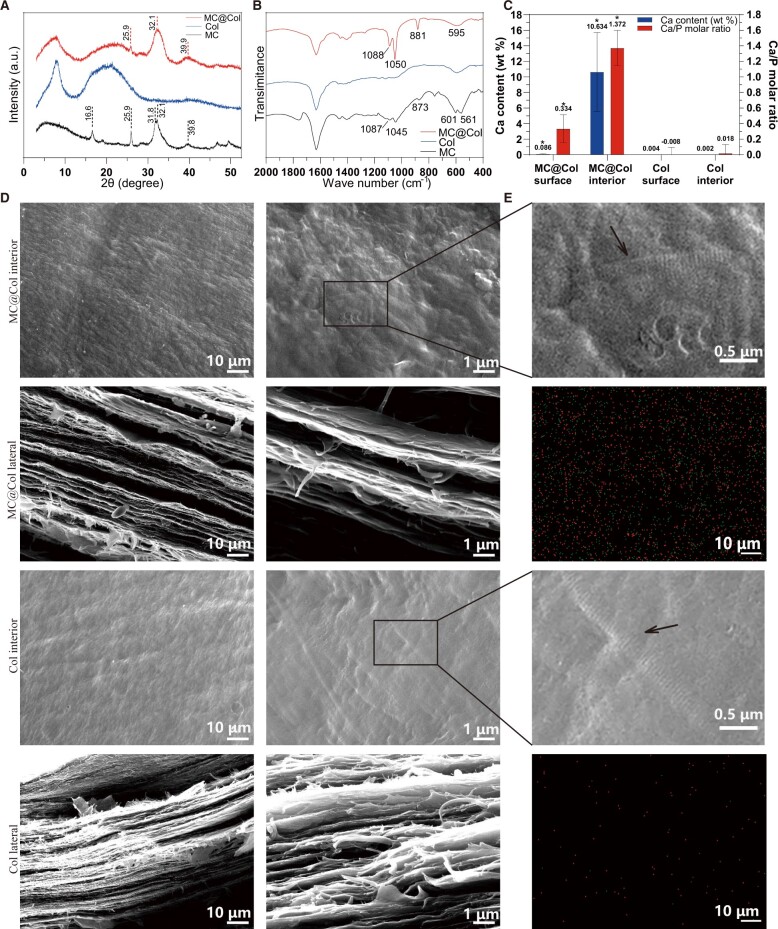
Molecular structures and surface morphologies of conduits. (**A**) XRD results of MC@Col, Col and MC. (**B**) FTIR results of MC @Col, Col and MC. (**C**) Quantitative statistical results of Ca and P elements on surfaces of MC@Col and Col through EDS, results are presented as the mean ± SD (*n* = 5; *P < 0.05). (**D**) SEM morphologies of superficial and lateral structures of MC @Col and Col in two magnifications. The lateral side showed a multi-layered structure. (**E**)The two former images showed the interior surface showed distinctive cross-band of collagen fibrils in MC@Col and Col, and it were magnified and marked in black arrow. The average period were calculated as nearly 64 nm and the diameter was nearly 200 nm in both groups. The latter revealed the distribution of Ca (green) and P (red) elements on inner surface of MC@Col (top) and Col (bottom), respectively. MC@Col, collagen-mineralized collagen nerve guidance conduit; Col, collagen nerve guidance conduit; MC, mineralized collagen; SD, standard deviation.

The XRD patterns, presented in Fig. 2A, showed the three different Col materials, including MC@Col, Col and MC. Three prominent crystalline peaks at 25.9°, 32.1° and 39.9° (2*θ*) were presented in the curve of the MC@Col scaffold, which were the distinctive diffraction peaks of HA crystals (PDF card no. 73-1731) and no peaks from other Ca–P materials were presented [25]. The diffraction peaks of the MC material presented two more peaks at 16.6° and 31.8° (2*θ*) as distinctive diffraction peaks of HA crystals [30], but without standard Col broad peaks. The Col group had the standard Col peaks at 7–8°and 20° (2*θ*) [36], but no obvious crystalline peaks were presented.

The FTIR spectra of MC@Col, Col and MC are shown in Fig. 2B, and the major peak locations of these materials were noted (see Supplementary Table S1). The spectra of the MC@Col and MC appeared as a superposition of calcium phosphate spectra and the Col fibrils spectra [25]. The spectra of the three materials exhibited the characteristic of peptide-based materials including amide A, B, I, II and III peaks, and these are related to the structure of Col [37]. The typical bands for Col such as N–H stretching for amide A (3400–3440 cm^−1^) [38] and C–H stretching for amide B (2956–3084 cm^−1^) [37] both had no shifting. The amide I peak (1628–1631 cm^−1^) [39], the amide II peak (1550–1600 cm^−1^) [40] and the amide III peak (1235–1238 cm^−1^) [41] had visualized in Col group. Based on previous study [25], in groups containing MC (MC@Col and MC group), a decrease of the intensity of amide I, II and III were observed, because the calcium phosphate crystals grow on the Col fibers and block the nucleation site, and then the peaks of amide II and III almost disappeared in this study. The peak positions are nearly identical for the MC fibrils and the precipitated calcium phosphate [25], but both were differing slightly from the values for pure HA. The spectroscopy of MC@Col showed the characteristic peaks (Col and calcium phosphate minerals), which verified that the calcium phosphate minerals were well distributed in MC@Col (Fig. 2B, Supplementary Table S1) [25].

**Figure 3.**
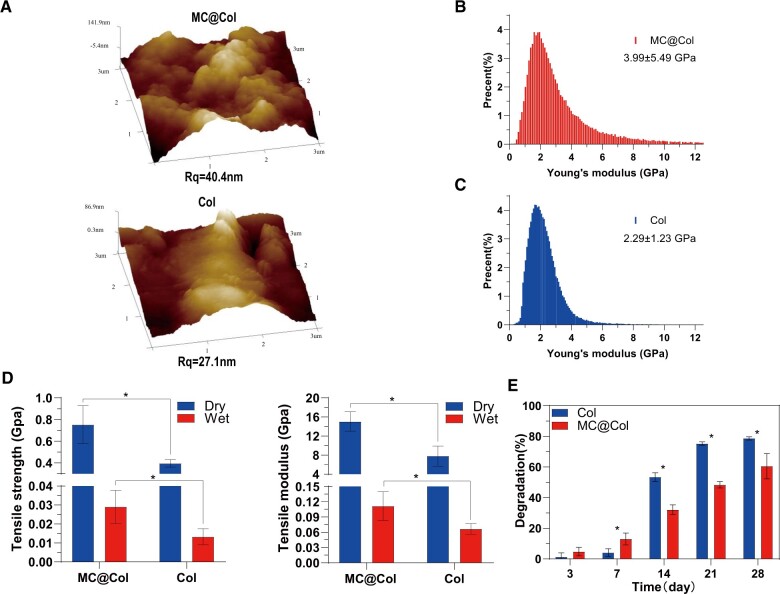
Physical properties and biodegradation properties of conduits. (**A**) AFM images of inner side of MC@Col and Col at 3 × 3 μm scan size. The surface of MC@Col materials (Rq = 40.4 nm) showed more roughness than the surface of Col membrane (Rq = 27.1 nm). (**B**) Stiffness distribution of MC@Col groups measured by QNM-AFM, and the Young’s modulus was 3.99 ± 5.49 GPa (mean ± SD). (**C**) Stiffness distribution of Col groups measured by QNM-AFM, and the Young’s modulus was 2.29 ± 1.23 GPa (mean ± SD). (**D**) Mechanical properties of MC@Col and Col, and results are presented as the mean ± SD (*n* = 5; *P < 0.05). (**E**) Biodegradation results of MC@Col and Col in 28 days *in vitro*, and results are presented as mean ± SD (*n* = 5; *P < 0.05). MC@Col, collagen-mineralized collagen nerve guidance conduit; Col, collagen nerve guidance conduit; MC, mineralized collagen; SD, standard deviation.

SEM and AFM were utilized to investigate the surface morphologies of different MC@Col and Col membranes (Figs 2D and 3A). Figure 2D showed the typical SEM images of the internal and lateral morphologies of the MC@Col and Col, which exhibited homogenous and compact appearances. Moreover, the fracture surface showed a multi-layered structure. Besides, it is noted that the typical Col fibrils with ∼ 200 nm in diameter and characteristic D-periodic cross-striated pattern (D = 64 nm) were observed in both membranes, indicating the good self-assembly of the Col molecules, as shown in Fig. 2D and E (black arrows) [30]. The MC@Col showed more roughness inner surface because of the existence of MC particles. The EDS element mapping profile confirmed the existence of MC (P in red and Ca in green) and their uniform distribution on the inner surface of the MC@Col membrane, but it almost not found in pure Col membrane (Fig. 2E). The Ca content was 10.63 ± 5.07 (wt %), and the mole ratio of Ca to P was 1.37 ± 0.23 (Fig. 2C). The EDS results were consistent with the XRD and FTIR results.

Moreover, the AFM examinations were used to show the roughness and stiffness distribution of the membranes. The inner surface of MC@Col membrane (Rq = 40.4 nm) showed more roughness than the surface of Col membrane (Rq = 27.1 nm), which has important influence on cell proliferation and differentiation [42]. Besides, the AFM scanning also provided the micro-mechanical properties of the membranes. The stiffness distribution profiles throughout the membranes were shown in Fig. 3B and C. The Young’s moduli of the MC@Col and Col membranes were 3.99 ± 5.49 GPa and 2.29 ± 1.23 GPa, respectively, indicating the involvement of MC was beneficial for the increase of the micro-mechanical property of the Col membranes. And the MC particles increased the surface heterogeneity, therefore the nano-stiffness of MC@Col was more widely distributed.

Figure 3D showed the macro-mechanical properties of MC@Col and Col membranes under dry and wet conditions. The tensile strength of the MC@Col membrane was significantly higher than that of the Col membrane both in dry conditions (0.75 ± 0.17 GPa versus 0.40 ± 0.03 GPa) and wet conditions (0.03 ± 0.01 GPa versus 0.01 ± 0.00 GPa), with the same trend found for Young’s modulus in dry conditions (15.04 ± 2.04 GPa versus 7.79 ± 2.10 GPa) and wet conditions (0.11 ± 0.03 GPa versus 0.07 ± 0.01 GPa). The MC could enhance the Young’s moduli of the Col scaffolds under both two conditions (*P *<* *0.05). In the tubular structure, the MC@Col conduits also showed a significantly higher strength in radial compressive resistance than Col and other conduits (Supplementary Fig. S1).

Figure 3E showed the *in vitro* biodegradation behavior of the different scaffolds. The results revealed that ∼ 80% of the Col scaffolds were degraded within 28 days, whereas 60% of the MC@Col scaffolds were degraded under the same conditions. After 14 days of degradation, the degradation rates of the MC@Col group were significantly slower than Col groups (*P *<* *0.05).

### *In vitro* results

#### Effect of MC@Col scaffold on RSC96 attachment and proliferation in vitro

**Figure 4.**
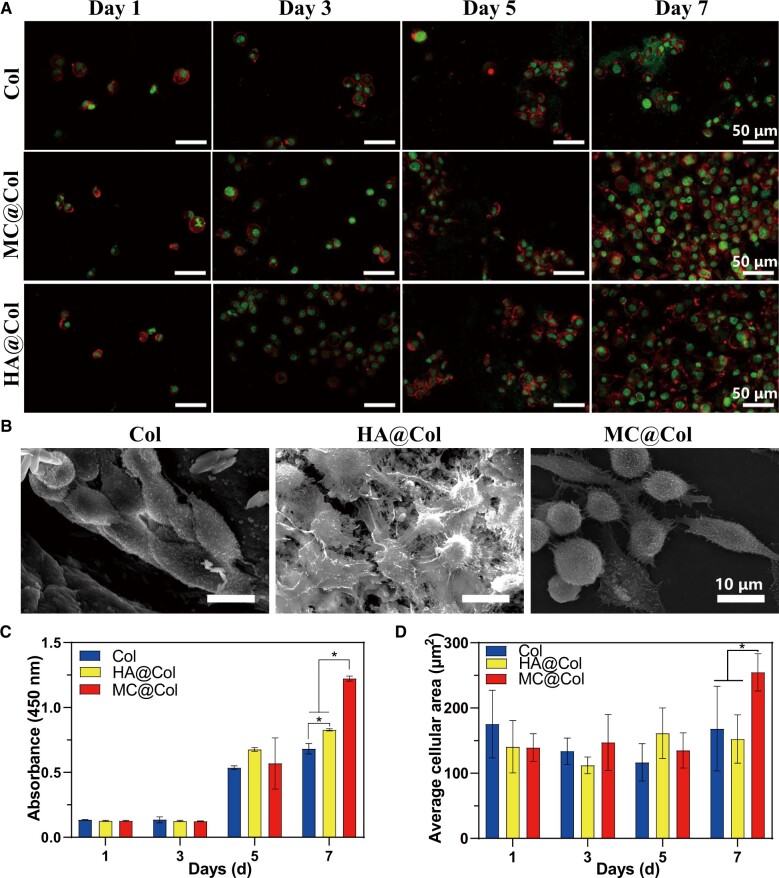
Morphology and proliferation of RSC96 on MC@Col, HA@Col and Col scaffolds. (**A**) Morphology of RSC96 on Days 1, 3 and 7 on MC@Col, HA@Col and Col scaffolds. Cells were stained with rhodamin-phalloidin for F-actin (red) and SYTOX green for nuclei (green). (**B**) Cell morphology on the surface of RSC96 on MC@Col, HA@Col and Col scaffolds observed via SEM. (**C**) Cell proliferation on Days 1, 3, 5 and 7. Results are presented as the mean ± SD; *n* = 5; *P < 0.05. (**D**) The average cellular area in five random fields (425 × 425 μm) on Days 1, 3, 5 and 7 on MC@Col, HA@Col and Col scaffolds, representing the spreading of co-cultured cell. Results are presented as the mean ± SD; *n* = 5; *P < 0.05.

The RSC96 were co-cultured with different scaffolds (MC@Col, Col and HA@Col) for 7 days, and the cell attachment and spreading were set as comparison. After 7 days, the cell densities on the MC@Col scaffold were higher than other two groups (Fig. 4A). The cell spreading was evaluated by calculating the average cellular area, and the single cell spreading area on the MC@Col scaffold was significantly larger than that on Col and HA@Col groups at 7 days (Fig. 4D), indicating that cell attachment was promoted in MC@Col. The SEM results also indicated the same results that cells in MC@Col group extruded more pseudopods (Fig. 4B). Therefore, the MC@Col was proved to promote RSC96 in adhesion and spreading than other groups.

There was no significant difference among all groups within 5-day culture. However, at 7 days, the proliferation rate of cells on MC@Col was significantly higher than that on Col and HA@Col (*P *<* *0.05), and there was a significant difference between the HA@Col and Col groups (*P *<* *0.05). Therefore, the MC@Col scaffold had no obvious cytotoxicity to hinder the nerve cell proliferation. Figure 4C showed that cell proliferation was tested by CCK-8 assay after 7 days of culture on various scaffolds.

### *In vivo* results

#### Morphological analysis of the regenerated nerves

**Figure 5.**
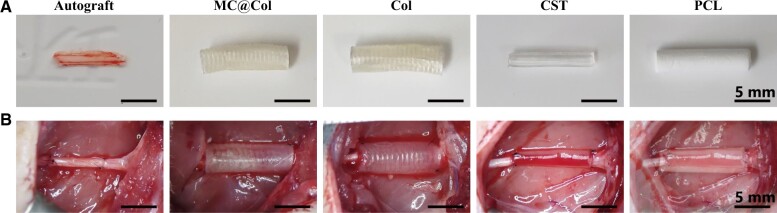
Preparation and implantation of the nerve grafts of the different groups. (**A**) Representative gross morphologies of autograft nerve grafting group (autograft), collagen mineralized collagen nerve guidance conduit group (MC@Col), collagen nerve guidance conduit group (Col), chitosan conduit group (CST) and polycaprolactone conduit group (PCL). (**B**) *In vivo* transplantation of each groups.

**Figure 6.**
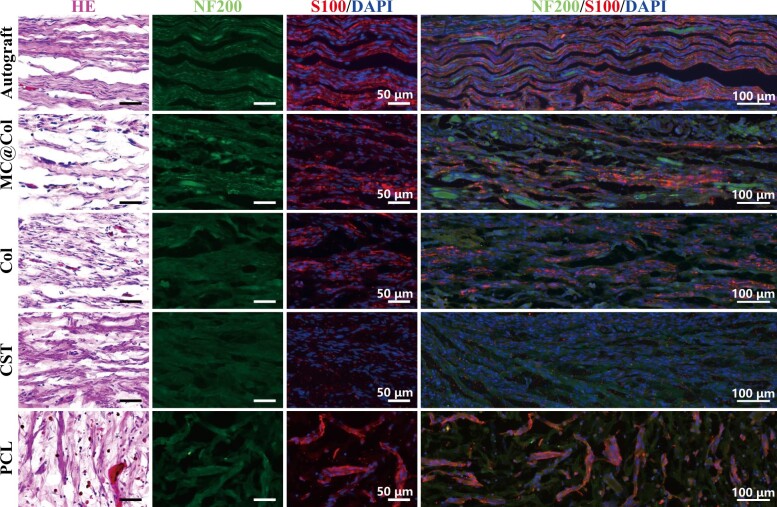
Hematoxylin–eosin (H&E) staining and immunofluorescence (IF) staining of the regenerated segments longitudinal following different groups within 12 weeks after surgery. H&E staining images were showed first column of five groups. The anti-neurofilament 200 antibody (NF200, a marker of nerve fiber; green) and anti-S100 antibody (a marker of Schwann cells; red) and DAPI (nuclear stain; blue) were stained before visualized by IF microscope, and the merged images of NF200/S100/DAPI labeling were also presented.

Four conduits (MC@Col, Col, CST, PCL) and autografts were used to bridge the sciatic nerve gap (Fig. 5), and the morphology results are shown in Fig. 6. First, there were no obvious inflammatory responses observed around the implants. Axons were observed in all five groups after 12 weeks postoperatively; however, there were differences on the density and direction of regenerating fibers. The NF200-stained cells in Autograft groups were arranged more regularly and longitudinal in comparison with that in the MC@Col groups, and the regenerated axons in the MC@Col and the Autograft groups were profuse and widely distributed. The other three nerve conduits (Col, CST and PCL) group, however, presented irregular and fewer regenerating axons. The images of S100- and DAPI-stained sections were merged (S100/DAPI, Fig. 6), and presenting a more pronounced S100 staining in the SCs in the MC@Col, Col and Autograft groups than in the CST and PCL groups. In the MC@Col and Autograft groups, abundant S100 staining were observed around the axons, suggesting that the MC@Col could promote the regeneration of axons and increase the number of SCs than the other conduits groups. In addition, the proliferating cells in PCL group were irregular, and did not extend longitudinally along with nerve axis.

**Figure 7.**
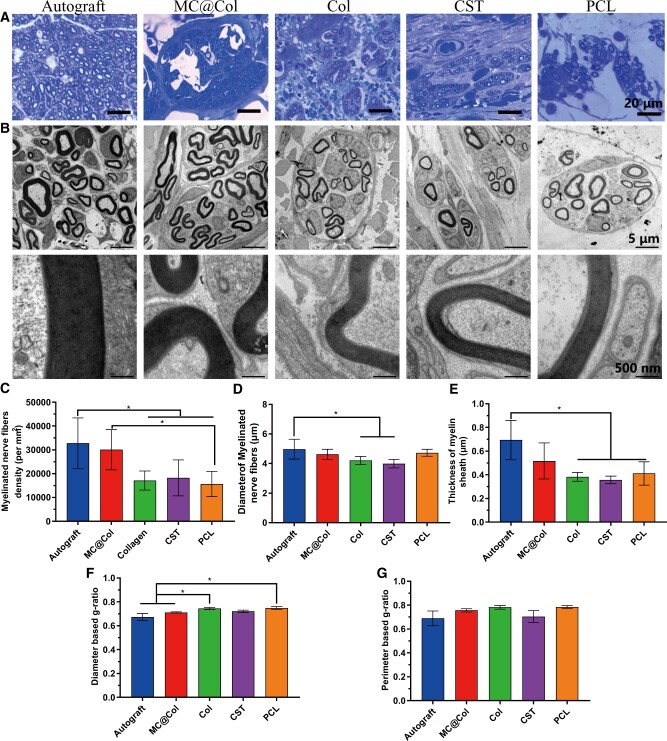
Morphometric analysis of the middle of the implants 12 weeks following different groups after surgery. (**A**) Toluidine blue-stained of semi-thin transverse sections. (**B**) Transmission electron micrographs of ultra-thin sections of the regenerated nerves. Histomorphometric analysis was performed by calculating the density of myelinated nerve fibers (**C**), the diameter of the myelinated axons (**D**), the thickness of the myelin sheath (**E**), diameter-based g-ratio (**F**) and perimeter-based g-ratio (**G**). Values are represented as mean ± SD (*n* = 5 per groups). *P < 0.05.

The cross-sectional slices of the middle part of the operated nerves after 12 weeks were observed both in an optical microscope and a TEM (Fig. 7). In the semi-thin sections (toluidine blue stained), uniform myelinated nerve fibers had grown across the middle of injured site in all groups; however, a denser packed nerve fibers were observed in the Autograft and MC@Col groups (Fig. 7A). The further quantitative results also proved this situation. The Autograft group (32 766.02 ± 10 686.39/mm^2^) showed significantly higher than the Col group (17 124.21 ± 4012.77/mm^2^), the CST group (18 248.78 ± 7507.42/mm^2^) and the PCL group (15 692.93 ± 5283.26/mm^2^) (*P *<* *0.05). In addition, the density of the nerve fibers of the MC@Col group (30 056.82 ± 8421.91/mm^2^) was significantly higher than the PCL group (*P *<* *0.05), and there was no significant difference between the MC@Col group and the Autograft group (*P *>* *0.05, Fig. 7C).

Through the TEM, similar results were observed from the ultra-thin sections **(**Fig. 7B). A compact and uniform regenerated axons beside the thick myelin sheaths were observed in MC@Col and Autograft groups at 12 weeks of after implantation. The diameter of myelinated nerve fibers calculated in the CST group (3.99 ± 0.30 μm) and the Col group (4.20 ± 0.28 μm) was significantly lower than that in the Autograft group (4.96 ± 0.67 μm) (*P *<* *0.05), and the MC@Col group (4.62 ± 0.34 μm) and the PCL group (4.72 ± 0.23 μm) were not significantly different with the Autograft group (Fig. 7D). The thickness of the myelin sheath in the Autograft group (0.69 ± 0.17 μm) was significantly larger than in the Col groups (0.38 ± 0.04 μm), the CST group (0.36 ± 0.03 μm) and the PCL group (0.41 ± 0.10 μm) (*P *<* *0.05). The sheath thickness of the MC@Col groups (0.52 ± 0.15 μm) was also not significantly different with that of the Autograft group (*P *>* *0.05), indicating better remyelination in these two groups (Fig. 7E).

As an evaluation parameter of the degree of fiber myelination, two types of g-ratio (diameter and perimeter based) were evaluated (Fig. 7F and G). The average values of diameter-based g-ratios/perimeter-based g-ratios in the Autograft group and the MC@Col group were all the nearest at 0.6, and no significant differences were detected between these two groups. The diameter-based g-ratios of the Col group (0.74 ± 0.01) and the PCL group (0.75 ± 0.02) were significantly different from those of the Autograft group (0.67 ± 0.03) and the MC@Col group (0.71 ± 0.00) (*P < *0.05). Furthermore, although the perimeter-based g-ratio were different within groups, there was no significant difference were detected between any two groups. According to the literature, the value of g-ratio of a normal peripheral nerve is found to approach 0.6 theoretically and experimentally [43]. Therefore, there was a better degree of myelination undoubtedly in the Autograft group and the MC@Col group because of their values of g-ratio [44].

#### Electrophysiological assessment

**Figure 8.**
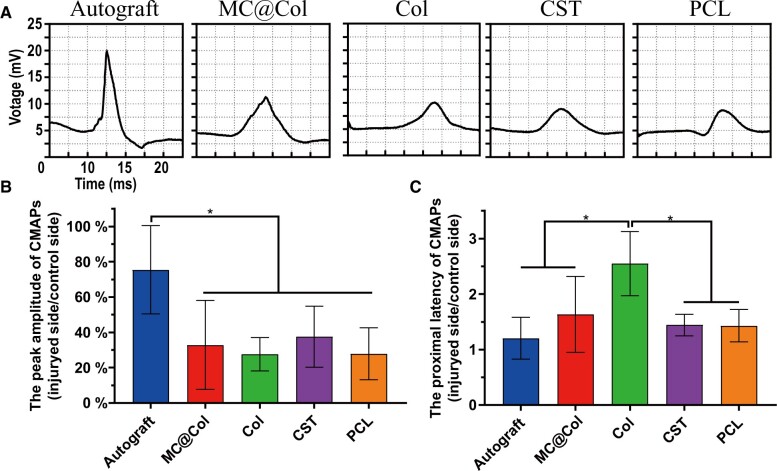
Electrophysiological evaluation of the nerve regeneration at 12 weeks post-implantation. (**A**) Representative electromyograph at the injured side in each group. (**B**) The peak amplitude of CMAPs (injury side/control side); *n* = 5 per group. (**C**) The proximal latency of CMAPs (injury side/control side); *n* = 5 per group. Values are represented as mean ± SD. *P < 0.05.

The recovery of nerve conduction was evaluated through the electrophysiological studies, which was represented by the changes of CMAP index in this study. According to literature, the amplitude and latency of CMAP reflects the number of innervated muscle fibers and the degree of axons myelination, respectively [34, 44]. Twelve weeks postoperatively, the representative CMAP curves of different groups was recorded and presented in Fig. 8A. Through this graph, both the CMAP latency of the MC@Col group and the Autograft groups were lower than the other three groups (the Col, CST and PCL groups), while the CMAP amplitude was also maintained differences obviously (Fig. 8A). The quantitative analysis was further performed to concretization the above results, and the ratio of CMAP index between the two sides was calculated and recorded in each group (Fig. 8B and C). The ratio of CMAPs amplitude in the MC@Col group (32.87 ± 25.16%) was lower than that in the Autograft group (75.39 ± 25.04%) significantly *(P *<* *0.05). In addition, there was no significant difference of CMAPs amplitude between the MC@Col group, the Col group (27.58 ± 9.52%), the CST group (37.52 ± 17.27%) and the PCL group (27.89 ± 14.68%) (*P *>* *0.05). Additionally, the CMAP proximal latency ratio of the MC@Col group (1.63 ± 0.68) was significantly shorter than that in the Col group (2.54 ± 0.58) *(P *<* *0.05), and there was no significant difference of CMAP latency between four groups, including the Autograft group (1.20 ± 0.37), the MC@Col group, the CST group (1.44 ± 0.19) and the PCL group (1.43 ± 0.29) (*P *>* *0.05) (Fig. 8C). Although the conductivities of regenerative nerve fibers were nearly consistent between conduits, there were also a trend of differences was observed. The recovery of nerve structure was slower than the function recovery may be a reason causing differences of morphological and electrophysiological results.

#### Reinnervated muscle weight and remodeling of muscle fibers

**Figure 9.**
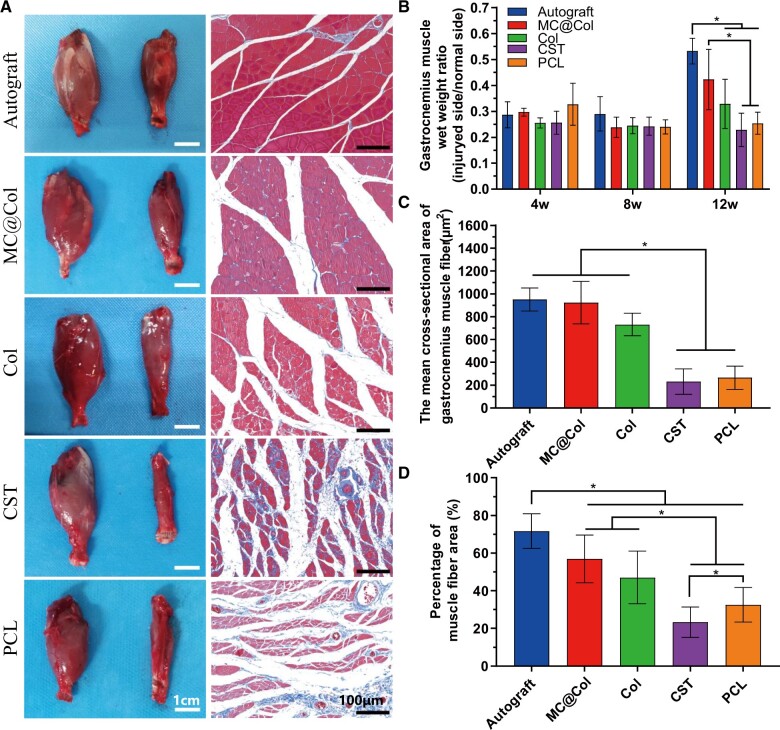
Morphometric analysis of gastrocnemius muscles after implantation with different groups. (**A**) Photographs of gastrocnemius and Masson’s trichrome staining images of the transverse sections of muscles from the injured limbs after 12 weeks of surgery. (**B**) Gastrocnemius muscle wet weight represented as the ratio between injured muscle wet weight relative to the uninjured muscle wet weight at 4, 8 and 12 weeks after surgery (*n* = 3 per group for 4 or 8 weeks; *n* = 5 per group for 12 weeks). (**C**) Cross sectional area of gastrocnemius muscle fibers at 12 weeks after surgery; *n* = 5 per group. (**D**) The percentage of muscle fiber area at 12 weeks after surgery; *n* = 5 per group. Values are represented as means ± SD. *P < 0.05.

The gross images and the stained slices images of gastrocnemius muscles at 12 weeks after implantation are shown in Fig. 9A. The muscle atrophy caused by denervation had not recovered completely in all groups but varied. There was an obviously gastrocnemius atrophy in the Col group, the CST group and the PCL group, and a relatively mild atrophy was seen in the target muscle in the MC@Col group and the Autograft group. The results of the muscle wet-weight ratio (Fig. 9B) confirmed that the MC@Col group (0.42 ± 0.12) and the Autograft group (0.53 ± 0.05) were significantly higher than those in the CST group (0.23 ± 0.06) and the PCL group (0.25 ± 0.04, *P < *0.05) in 12 weeks. Neither the Col group nor the Autograft group had significant difference with MC@Col group (*P *>* *0.05). The results differences of the muscle wet-weight ratio in groups were partly consistent with the gross observation results. Seeing the cross-section images from different groups, the muscle fibers in the PCL groups and the CST groups were degenerated heavily and confirmed a considerable atrophy. A large amount of the Col deposition (blue in the images) was observed in the Col group, the CST group and the PCL group; however, little Col were seen in the MC@Col group and the Autograft group. The cross-sectional area of gastrocnemius muscle fibers in the MC@Col groups (923.30 ± 187.09 μm^2^) was significantly higher than that from the CST group (230.94 ± 111.16 μm^2^) and the PCL group (265.75 ± 101.75 μm^2^) (*P *<* *0.05), but similar to the Autograft group (951.06 ± 100.43 μm^2^) and the Col group (731.78 ± 96.70 μm^2^, *P *>* *0.05, Fig. 9A and C). Compatible with above results, the percentage of muscle fiber area (calculated from the staining images) also confirmed a better recovery in the MC@Col group and the Autograft group (Fig. 9D).

#### CatWalk gait analysis

A complete sciatic nerve injury leads to a morbid walking, such as a tendency to drag the injured side foot, a deficiency of plantar flexion of the ankle and less ankle spreading [45]. When the injured gap of sciatic nerve regenerates, the moto function will return partially.

**Figure 10.**
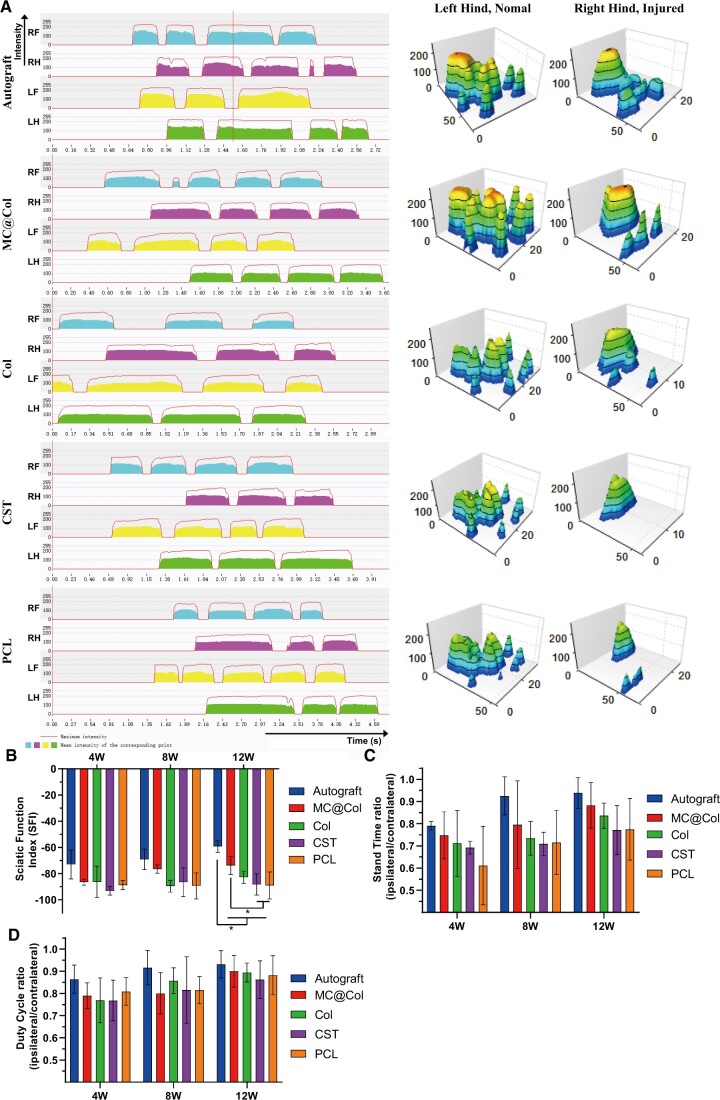
Motor function recovery as examined by CatWalk gait analysis 12 weeks after surgery. (**A**) The 2D foot intensities over time and the 3D plantar pressure distributions of the hindlimbs of both the injured and contralateral sides of the five groups. The *x*-axis represents the time, and the *y*-axis represents the intensity of the limbs of the tested rats. RF, right front limb; RH, right hindlimb; LF, left front limb; LH, left hindlimb. The color blocks of RH showed shorter time to attach the ground and lower intensity when touch the ground in 2D foot picture. (**B**) The SFI values of different groups. (**C**) The stand time ratios and (**D**) Duty cycle ratio of different groups. Values are represented as mean ± SD (*n* = 5 per group in 12 weeks; *n* = 3 per group in 4 or 8 weeks, respectively). *P < 0.05.

A qualitative indication of motor function was first tested. The *x*-axis and the *y*-axis of was represented time and limb intensities, respectively, in the 12 weeks postoperative 2D graphs (left of Fig. 10A). The right hind (RH, injured) is shorter than that of the left hind (LH, normal), showing that the injured limb had not fully recovered with shorter touch time and lower intensity. The three-dimensional graphs showed the plantar pressure distribution of the RH and LH paws (right side of Fig. 10A). The plantar pressure and the toe spread in the MC@Col groups was obviously similar to that of the Autograft group and better than those in the other groups. These indicated that the MC@Col group was better in motor function recovery.

The SFI value is a quantitative indicator for evaluating the nerve dysfunction and recovery (Fig. 10B), while –100 indicates a complete dysfunction and 0 represents normal function [34]. The SFI values of the MC@Col groups displayed significant differences compared with those in the CST and PCL groups after 12 weeks *(P < *0.05), but no significant difference in 4 and 8 weeks after surgery (*P *>* *0.05). In addition, values of the MC@Col group were the closest to those of the Autograft group after 12 weeks (Fig. 10B). An impaired motor function caused by nerve defect resulted in the decrease of SFI values in initial time, and then the SFI values increase as a result of nerve regeneration [46]. It is noted that the SFI values of the Autograft group and the MC@Col group showed early improvement from fourth week in a time-dependent manner, while other groups showed either no obvious improvement (PCL group) or a slower recovery (Col and CST group). In addition, as the closest to the Autograft group, the SFI values in the MC@Col group displayed nearly synchronous trend through the whole period (see Supplementary Fig. S2). These results suggested that the MC@Col conduit could improve the functional recovery of sciatic nerve after injury.

There were no significant differences on the stand time ratio (RH/LH) among different groups in each post-operational time (*P *>* *0.05, Fig. 10C). The stand time ratio descended rapidly after the first 4 weeks; however, an uprising trend was observed with different efficiency (see Supplementary Fig. S3). The stand time ratio of Autograft group had a least decline and the fastest recovery, followed by the MC@Col group. Other groups (Col, CST, PCL group) had a relatively poor result than the contralateral hind paw but without significant differences. No significant differences were found in the duty cycle ratio among all groups in 4, 8 and 12 weeks (*P *>* *0.05, Fig. 10D). The recovery efficiency of Col group was higher in first 8 weeks but dropped after then. On the contrary, the MC@Col group recovered slower initially but improved after 8 weeks (see Supplementary Fig. S4).

## Discussion

Col has a widespread use as a typical biological material in peripheral nerve repair because of its weak antigenicity and good biocompatibility for cell growth and transportation of nutrients [47–51]. However, poor mechanical and inappropriate degradation properties limit the utilization of Col in peripheral nerve regeneration [10]. The MC that was prepared by a biomimetic *in situ* mineralization process has good cytocompatibility and wide applications in bone regeneration [23, 24, 52]. Considering the positive effect of calcium ions on nerve regeneration, the MC was applied for the first time in peripheral nerve repair. In this study, a Col-based conduit, MC@Col, was prepared by adding the MC to Col matrix, aiming to improve the degradation, mechanical properties and further biological activity of this conduit.

The MC distributed uniformly on the surface of the Col membrane, which contributed to the increased surface roughness and mechanical properties. The involvement of MC showed positive effects on SCs attachment and proliferation *in vitro*. Through *in vivo* implantation, the MC@Col showed a good result and acted as the closest one with the Autograft group in comparison with other conventional NGC materials including Col, CST and PCL. The regenerated axons of the MC@Col group filled in the gap with an aligned structure and reached the distal stump at 12 weeks after surgery. The qualitative and quantitative evaluations indicated that the nerve fiber density, nerve fiber diameter and myelin sheath thickness of the MC@Col group were close to Autograft group. Although the electrophysiological result in the MC@Col group was not as good as that in the Autograft group, the target muscle evaluation and behavioral analysis further showed a similar result between the autograft and the MC@Col. The *in vivo* evaluation implied that the MC in the MC@Col group was beneficial to axon regeneration and myelination, which further contributed to the reinnervation of target muscles and motor functional recovery.

Col, as a typical biological material, has widespread biomedical applications including peripheral nerve repair [48, 50, 51]. Although the mechanical properties and degradation rate could be ameliorated by many physical and chemical crosslinking methods [53], they are still not satisfactory especially in wet state. In this study, it is the first time to add MC in Col substrate for peripheral nerve regeneration. Unlike pure HA, MC is a co-assembling composite of Col and nano-HA, in which HA is chemically bound to Col. Thus, nano-HA distributed uniformly on the surface of the MC@Col membrane and kept strong chemical interaction with Col, which may enhance the mechanical properties of the membrane. Besides, after rolling into tube, the MC was distributed between layers that also was favorable to the improvement of mechanical properties.

Another advantage is the sustained release of calcium ions from the MC@Col. Although calcium ion has been recognized as an important signaling factor for the nerve regeneration [17, 18, 54], few studies had used it in nerve conduit. Chen *et al*. [55] invented a dexamethasone-releasing poly-D-L-lactide/nano-HA composite bone scaffold, and the results show that HA can slow down the degradation and mass loss in scaffold. Based on this feature, Wu *et al*. [56] used HA as an ingredient to develop a nerve conduit for a 10-mm rat sciatic nerve repair, which turned out a good result. Nawrotek *et al*. [57] also invented a calcium ion-containing CST–carbon nanotube nerve implant, which could coordinate growth cone movement by controlling the concentration of Ca^2+^. In another study, researchers used a HA nanoparticle-containing Col hydrogel in the regeneration of sciatic nerve crush injury, which also confirmed the positive effects of Ca^2+^ on SCs proliferation and axonal outgrowth [19]. Therefore, the calcium ions that released from the MC@Col may exert good biological activity on promoting injured nerve repair. In MC, the nano-HA nanoparticles deposited on the specific sites of the Col fibrils with the chemical interactions between the calcium ions of HA and the carboxyl and carbonyl groups on Col, which will contribute to the sustained release of the calcium ions accompanying with the MC degradation. Therefore the MC@Col group showed better performance than HA@Col group with appropriate biocompatibility and calcium release [58].

The MC@Col tube combined with excellent physical performance, biocompatibility and biological effects, creating the most desirable effects for axon regeneration compared with other conduits (CST, PCL and Col). However, it still had a difference with the Autograft. As a hollow NGC, the internal microenvironment needs to be improved through matrices filling in the lumen. In our group, an aligned fibrin nanofiber hydrogel has been used for accelerating peripheral nerve regeneration as an excellent intraluminal filling [34], which will be combined with the MC@Col conduit in the following study for further improving nerve regeneration. Besides, the cellular and molecular mechanism of the effects of the MC on regulating nerve regeneration was still not clear, which need further investigations.

## Conclusions

In this study, a MC-based NGC was developed and evaluated in its repair performance in PNI both *in vitro* and *in vivo*. The MC@Col showed enhanced mechanical properties and biodegradation properties comparing the general material. *In vitro* study indicated that the MC@Col could promote the attachment and alignment of SCs, facilitated nerve repair. Additionally, the MC@Col tubes implantation *in vivo* accelerated axonal regrowth, improved histological morphogenesis and improved motor functional recovery than other sample groups. In conclusion, it was a promising potential that MC@Col conduit could highly advancing the peripheral nerve regeneration.

## Supplementary data

Supplementary data are available at *Regenerative Biomaterials* online.

## Ethics approval and consent to participate

This study was approved by the ethics committee of Laboratory animal research center of Tsinghua University (Approval document number: 19-WXM2). All the procedure about animals were carefully carry out based on the standard guidelines.

## Consent for publication

Not applicable.

## Availability of data and material

The datasets generated during and/or analyzed during the current study are available from the corresponding author on reasonable request.

## Funding

This work was funded by the National Key R&D Program of China (No. 2020YFC1107601), The Foshan-Tsinghua Innovation Special Fund (No. 2020THFS05) and the Key R & D Program in Shandong Province (2019JZZY011106).

*Conflicts of interest statement.* The authors declare that they have no conflict of interest.

## Authors’ contributions

G.D. conceived of the manuscript and participated in data collection, data analysis, and preparation of manuscript; C.L. and X.Y. participated in data collection and data analysis; S.Y., S.W., X.S., L.Z. and T.S. participated in data collection and data analysis; Y.P. and X.W. were corresponding author, supervised, co-ordinated and provided further advice on revisions. All authors read and approved the final manuscript.

## Supplementary Material

rbac089_Supplementary_Data
