# Supplementary material for: Construction of a mineralized collagen nerve conduit for peripheral nerve injury repair
Source: Regen Biomater. 2022 Oct 31;10:rbac089. doi: 10.1093/rb/rbac089 (PMC9847629; doi:10.1093/rb/rbac089)
Supplement: rbac089_Supplementary_Data [file rbac089_supplementary_data.docx]

**Additional File**

**Table S1. Fourier-transform infrared spectral** **peak locations and assignments for MC@Col, Col and MC**

|  | Peak wavenumber (cm^-1^) | | | | Peak region assignments |
| --- | --- | --- | --- | --- | --- |
|  | MC@Col | Col | MC | Phosphate ^a^ |  |
| Amide A | 3424 | 3431 | 3429 | - | N-H stretching of proteins coupled with hydrogen bond |
| Amide B | 2973 | 2956 | 2972 | - | Amide B:CH_3_-asymmetrical stretch |
| Amide I | 1631 | 1631 | 1630 | - | C=O stretch/hydrogen bond coupled with COO^-^ |
| Amide II | - | 1502 | - | - | N-H bending/C-N stretching of proteins |
| Amide III | 1273 | 1238 | 1275 | - | N-H bend coupled with C-N stretch (β-sheet, protein) |
| Phosphate Bands | 1088 | - | 1087 | 1089 | HPO_4_^3-^ (ν_3_) |
|  | 1050 | - | 1045 | 1050 | HPO_4_^3-^ (ν_3_) |
|  | 881 | - | 873 | 875 | HPO_4_^2-^ (ν_4_) |
|  | 595 | - | 601 | 601 | HPO_4_^2-^ (ν_4_) |
|  |  | - | 561 | 562 | HPO_4_^2-^ (ν_4_) |

^a^ Peak Positions of the Phosphate Bands in Calcium Phosphate and HAP particles were from previous studies [26,43]

(Zhu et al. 2020; Zhang et al. 2003)

**Figure S1. The radial compressive resistance behavior of different conduits.** (A) The compressive force corresponding to the diameter of conduits reduced by 50% in different groups (n = 3 per group); Values are represented as mean ± SD. **p* < 0.05. (B) Representative cross-section views of the conduits after compression.


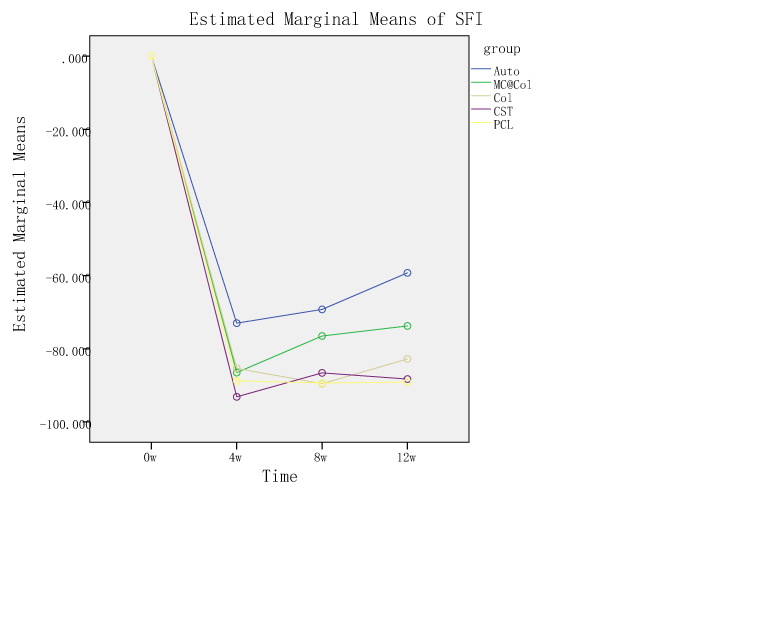


**Figure S2. The estimated marginal means of SFI values at different post-operational time in different groups.** The Autograft and MC@Col groups showed early and efficient improvement after fourth week in a time-dependent manner. The other three hollow conduit groups not only had a lower SFI values but also with inefficiency after four weeks, The SFI values of COL group recover until 8 weeks, and the CST group had an instability recovery, while the PCL group showed no obvious improvement.


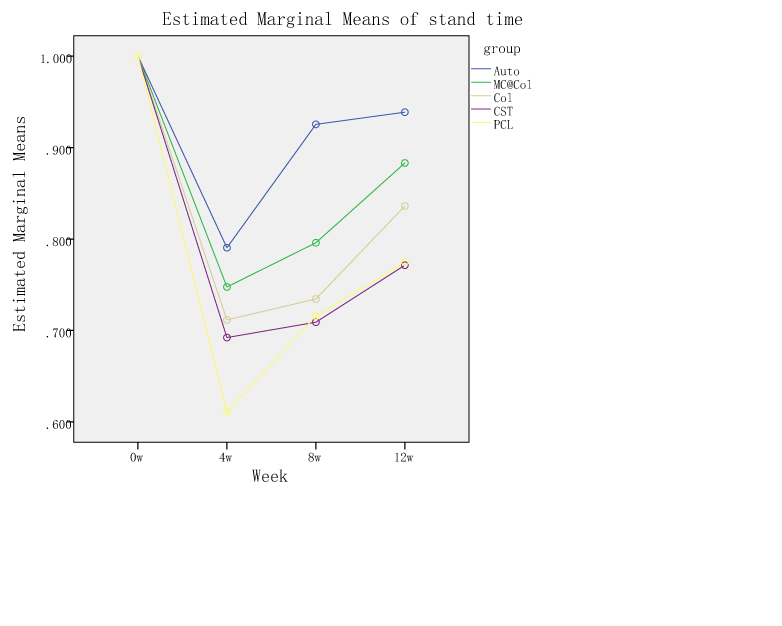


**Figure S3. The estimated marginal means of stand time ratio at different post-operational time in different groups.** The stand time ratio (RH/LH) descends rapidly after the first 4-week, however, the uprising was observed with different efficiency. The stand time ratio of Autograft group had a least decline and the fastest recovery, followed by MC@Col group. The other group (Col, CST, PCL group) had a relatively poor result than the contralateral hind paw but without significant differences.


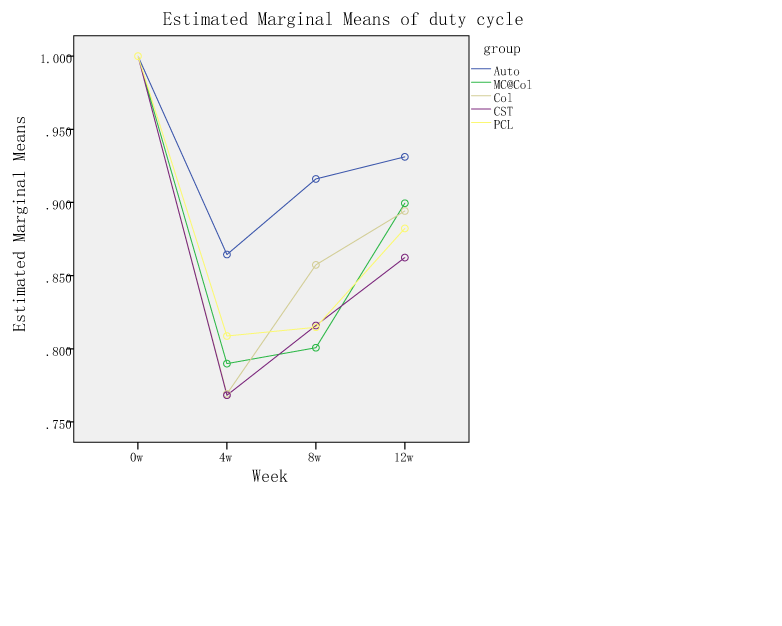


**Figure S4. The estimated marginal means of duty cycle ratio at different post-operational time in different groups.** The recovery efficiency of Col group was higher in first 8-week but dropped after then. On the contrary, the MC@Col group recovered slower initially but improved after 8 weeks.
